# HP0197 Contributes to CPS Synthesis and the Virulence of *Streptococcus suis* via CcpA

**DOI:** 10.1371/journal.pone.0050987

**Published:** 2012-11-30

**Authors:** Anding Zhang, Bo Chen, Zhengzhi Yuan, Ran Li, Cheng Liu, Hongbo Zhou, Huanchun Chen, Meilin Jin

**Affiliations:** 1 Unit of Animal Infectious Diseases, National Key Laboratory of Agricultural Microbiology, Huazhong Agricultural University, Wuhan, Hubei, People's Republic of China; 2 College of Veterinary Medicine, Huazhong Agricultural University, Wuhan, Hubei, People's Republic of China; University of Kansas Medical Center, United States of America

## Abstract

*Streptococcus suis* serotype 2 (SS2), a major swine pathogen and an emerging zoonotic agent, has greatly challenged global public health. The encoding proteins with unknown functions the bacterium encodes are an obstruction to studies of the pathogenesis. A novel surface protective antigen HP0197 is one of these proteins which have no sequence homology to any known protein. In the present study, the protein was determined to be involved in bacterial virulence through an evaluation of the isogenic mutant (Δ*hp0197*) in both mice and pigs. The experimental infection also indicated that Δ*hp0197* could be cleared easily during infection, which could be attributed to the reduced thickness of the capsular polysaccharides (CPS) and the significantly reduced phagocytotic resistance. Microarrays-based comparative transcriptome analysis suggested that the suppressed expression of the operon responsible for CPS synthesis might be reversed by CcpA activity, which controlled global regulation of carbon catabolite through the binding of the CcpA and HPr-Ser-46-P to the catabolite-responsive elements (*cre*) of the target operons. The hypothesis was approved by the fact that the purified FLAG-tagged HPr from WT stain exhibited a higher binding activity to *cre* with CcpA compared to the Δ*hp0197* by the Electrophoretic Mobility Shift Assay, suggesting lower level of phosphorylation of the phosphocarrier protein HPr at residue Ser-46 (HPr-Ser-46P) in Δ*hp0197.* These indicated that HP0197 could enhance CcpA activity to control the expression of genes involved in carbohydrate utilization and CPS synthesis, thus contributing to the virulence of *S. suis*.

## Introduction

*Streptococcus suis* (*S. suis*) is a major swine pathogen responsible for severe economic losses in the porcine industry. It is also a severe threat to human health, especially to people who are in close contact with swine or pork by-products. *S. suis* serotype 2 (SS2) is considered the most pathogenic and the most prevalent capsular type among the thirty-three serotypes (types 1 to 31, 33, and 1/2) [1], [2], [3]. The infection caused by *S. suis* has been reported in more than 20 countries, in which more than 700 people have been infected since the first reported case of *S. suis*-caused human meningitis in Denmark in 1968 [4]. Two recent large-scale outbreaks of human SS2 epidemics in China (the first had 25 cases with 14 deaths in Jiangsu in 1998, and the second had 204 cases with 38 deaths in Sichuan in 2005) have raised people’s awareness of the public health threat because the cases presented clinically with a streptococcal toxic shock-like syndrome, which indicates that new virulent bacterial variants are currently emerging in Asia [5], [6], [7], [8], [9], [10], [11]. Besides, the bacteria have also caused sporadic human illness in other countries [12], including Thailand, the United Kingdom, Portugal, Italy, Japan, Australia, Netherlands and the United States. *S. suis* has also been identified as the third most common cause of community-acquired bacterial meningitis in Hong Kong and as the leading cause of adult meningitis in Vietnam [7], [13], [14]. The infected cases have occasionally been documented in North America, and some investigators believe that more cases could have happened than what have been reported, and the relatively low number of reported cases of human *S. suis* infections is hypothesized to be a result of misdiagnosis rather than the true absence of the disease [15], [16].

Herein, *S. suis* infection has attracted a great deal of attention from the scientific community and the popular press [17]. However, the current understanding of the *S. suis* pathogenesis is still limited. The polysaccharide capsule has generally been considered to be essential for bacterial virulence [18], [19], [20]. Suilysin, the extracellular protein factor, along with a muramidase-released protein have also been shown to be linked to, but not essential for, the full virulence of *S. suis* [21]. GapdH [22], Enolase [23], [24], Fibronectin/Fibrinogen-binding protein [25], HAM1 [26] and Adhesion [27], [28], [29], [30], [31], [32] have been shown to be involved in *S. suis* adherence and virulence. Serum opacity-like factor [33], D-Alanylation of Lipoteichoic Acid [34], Peptidoglycan GlcNAc deacetylase [35], IgA protease [36], [37], TroA [38], SodA [39], SsFHB [40] and Subtilisin-like serine protease [41], [42], [43] are also considered to be related to *S. suis* virulence. In addition, studies have reported that SalK/SalR [44] and CovR [45] affected the virulence of Chinese isolates of *S. suis*. Although series virulence-associated factors have been discovered, which helped people understand *S. suis* pathogenesis, the infection process related to the disease remains unknown.

Genomics and proteomics studies have revealed that there are several hypothetical proteins on the surface of the bacterium [9], [46], [47], [48], [49], [50]. Among these, HP0197 (SSU98_0197 in strain 98HAH33 or SSU05_0196 in strain 05ZYH33) has an YSIRK-type signal peptide presented at the N terminus and a typical C-terminal sorting signal of “LPATG” motif and is located on the bacterial surface [48], [50]. Our previous study indicated that HP0197 presented in all tested clinical SS2 isolates and was identified as a surface protective antigen which could confer significant protection against challenge with lethal dose of SS2 in mice and pigs [51]. All of these information indicated that the protein may be involved in the pathogenesis of *S. suis*. However, this hypothetical protein has no similar domain architectures except for a G5 domain (pfam07501) which has been identified in several proteins involved in the metabolism of the bacterial cell walls [52]. Therefore, it is not easy to predict the function of the protein by bioinformatics analysis because only 76 aa (418–493 aa) out of 562 aa are found to be similar to the G5 domain; the rest of this protein is not similar to any known protein domain. Thus, evaluation on the contribution of HP0197 to the virulence would strengthen the understanding of this protein, and undoubtedly be conductive to the understanding of the pathogenicity of the *S. suis*.

## Results

### Construction of Δ*hp0197*

**Figure 1:**
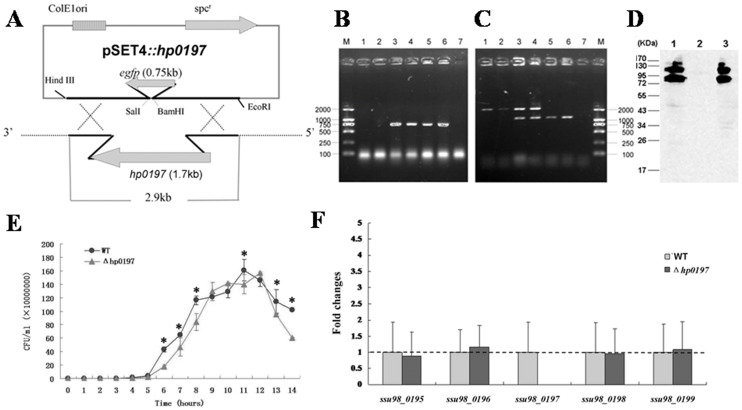
Construction and confirmation of Δ*hp0197* and a complementary strain, cΔ*hp0197*. (**A**) Construction strategy for Δ*hp0197*, which was derived from the SS2 strain 05ZY. Approximately 600 bp of sequence flanking *hp0197* was constructed into the temperature-sensitive *S. suis*-*E. coli* shuttle vector pSET4s, *hp0197* was replaced with *egfp*. (**B**) The *egfp* gene could not be amplified from the WT strain (lane 1 and 2) but was amplified from the Δ*hp0197* (lane 5 and 6) and cΔ*hp0197* strains (lane 3 and 4). (**C**) A PCR targeted to the 176 bp and 252 bp from the upstream and downstream sequence of *hp0197* sequence respectively with primer pairs of HP0197-P1/HP0197-P2 was performed to confirm the successful construction. And a 2.1-kb band could be amplified from DNA of the WT strain (lanes 1 and 2), and a 1.2-kb DNA fragment could be amplified from the Δ*hp0197* mutant (lanes 5 and 6). Two bands were amplified from the cΔ*hp0197* strain (lanes 3 and 4). Lane 7 in A and B were PCR negative controls. (**D**) HP0197 antibodies did not bind proteins extracted from the Δ*hp0197* mutant strain (lane 2) but was able to bind proteins extracted from the WT strain (lane 1) and the cΔ*hp0197* strain (lane 3) in the immunoblot assay. (**E**) The growth rates of *S. suis* strains in THB. (**F**) qRT-PCR amplification of *hp0197* (*ssu98_0197*) flanking genes (*ssu98_0195* to *ssu98_0199*) yielded identical results in WT and Δ*hp0197* while *hp0197* could only be amplified from WT.

To study the role of HP0197 in *S.suis* virulence, an isogenic *hp0197* mutant was derived from strain 05ZY (**Fig. 1A**). In Δ*hp0197* mutant, *hp0197* gene (1.7 kb) was replaced with *egfp* gene (0.75 kb). So PCR with primer pairs of HP0197-P1/HP0197-P2 which targeted to the 176 bp and 252 bp from the upstream and downstream sequence of *hp0197* gene respectively could be used to confirm the deletion of *hp0197* gene. A 2.1 kb or 1.2 kb DNA band was obtained respectively when the genome DNA of WT or Δ*hp0197* strain served as a template. Besides, an *egfp* gene could not be amplified from the genome of WT strain but from the constructed Δ*hp0197*. These tests indicated that *hp0197* gene was successfully replaced by *egfp* gene **(** **Fig. 1B and 1C** **)**. HP0197 antibodies could react with proteins extracted from WT and cΔ*hp0197* (complemented strain of Δ*hp0197*) rather than from Δ*hp0197* in the immunoblotting assay, which indicated no expression of HP0197 in Δ*hp0197* and the successful complementation for cΔ*hp0197* strain **(** **Fig. 1D** **)**.

Δ*hp0197* showed slower growth rate than WT **(** **Fig. 1E** **)**, which provoked us to illustrate whether this phenomenon was resulted from the polar effect when *hp0197* gene was deleted. The expression of the *hp0197* flanking genes (*SSU98_0196* and *SSU98_0198*) was not changed significantly in WT and Δ*hp0197* by qRT-PCR, which further indicated that the deletion of *hp0197* could not cause obvious polar effect **(** **Fig. 1F** **)**. These results suggested that the deletion of *hp0197* may retard the growth rate of *S. suis* at given time course.

### HP0197 Contributes Significantly to *S. suis* Virulence in Mice

**Figure 2:**
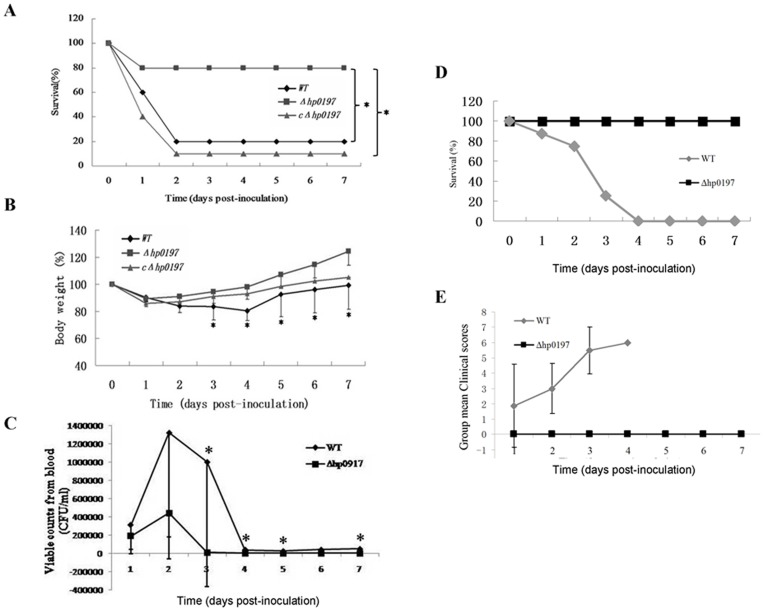
HP0197 contributes significantly to *S. suis* virulence in mice and pigs. (**A**) The survival of experimental mice inoculated with WT, Δ*hp0197* and cΔ*hp0197* strains. Ten mice in each group were inoculated by intraperitoneal injection with 10^9^ CFU of WT, Δ*hp0197* or cΔ*hp0197* respectively and the mice were monitored for clinical signs of infection three times a day during 7 days. “*” represents P value <0.05 of WT versus Δ*hp0197* group *or* Δ*hp0197* versus *c*Δ*hp0197* group *respectively.* (**B**) The loss of body weight of mice inoculated with the WT, Δ*hp0197* and cΔ*hp0197* strains, which usually reflects the health status of mice. “*” represents P value <0.05 of WT versus Δ*hp0197* group. (**C**) The kinetics of bacterial clearance from the blood of mice inoculated with the WT, Δ*hp0197* and cΔ*hp0197* strains. “*” represents P<0.05 of WT versus Δ*hp0197* group at given time course. (**D**) The survival of experimental pigs inoculated with WT and Δ*hp0197* strains. Eight pigs in each group were inoculated intranasally with 2×10^7^ CFU of WT and Δ*hp0197* strains respectively. Four pigs were inoculated with PBS and served as a control. “*” represents P<0.05 of WT versus Δ*hp0197* group. (**E**) Clinical scores (daily means and standard deviations) of pigs after inoculation. “*” represents P value <0.05 of WT versus Δ*hp0197* group at given time course.

HP0197 has been identified as an important vaccine candidate that exists in almost all tested pathogenic clinical SS2 isolates [51]. Therefore, it is worth evaluating its role in *S. suis* virulence. Independent experimental infections with the wild-type (WT), Δ*hp0197* and cΔ*hp0197* were performed in mice **(** **Fig. 2** **)**. While all mice manifested depression, weakness and prostration during the first day post-inoculation (pi), the mice in the WT and cΔ*hp0197* groups showed more severe clinical signs. Eighty percent of the mice in the WT group and 90% of the mice in the cΔ*hp0197* group died within the first 2 days pi. After 2 days, the remaining mice recovered from serious clinical symptoms. In contrast, only 2 mice in the Δ*hp0197* group died on day 1; the rest of the mice survived during the trial **(** **Fig. 2A** **)**. These indicated that the deletion of *hp0197* decreased the lethality of SS2 to mice (*P*<0.05). The loss of body weight usually reflects upon the health of the mice, and the body weight of mice in Δ*hp0197* group recovered more rapidly than mice in WT group since day 3 p.i. (p<0.05) **(** **Fig. 2B** **)**, which further indicated that the deletion of *hp0197* decreased the pathogenicity of SS2 to mice.

The resistance to clearance from circulation could reflect the evasion of pathogen from host defense system. The level of recovered bacterial in each group did not show significant difference on day 1 p.i., and the bacterial reached the highest level on day 2 p.i. **(** **Fig. 2C** **)**. However, the recovered SS2 in Δ*hp0197* group decreased more rapidly on day 3 p.i. than WT group (p<0.05). Furthermore, no bacterium could be isolated from circulation of Δ*hp0197* group since day 4 p.i. while bacteria still sustained in the blood of mice in WT group or in cΔ*hp0197* group. These indicated that Δ*hp0197* was easily cleared by the host **(** **Fig. 2C** **)**.

This trial revealed that compared to the WT strain, Δ*hp0197* was significantly attenuated and that the reintroduction of the *hp0197* gene into Δ*hp0197* (cΔ*hp0197*) restored high levels of pathogenicity to the bacterial infection. The experimental infection in the mice strongly suggested that HP0197 played an important role in the pathogenesis of SS2 to mice.

### Infection with the Δ*hp0197* is Significantly Attenuated in Pigs

To further delineate the role of *hp0197* in *S. suis* virulence, we conducted a trial in pigs, which are the natural hosts of infection. Four pigs in the sham-inoculated group did not manifest any clinical signs of infection during the test. Conversely, all of the pigs in the WT group (n = 8) died within 3 days **(** **Fig. 2D** **)**, and they all showed severe clinical signs of disease, including severe respiratory symptoms, lameness associated with swollen joints and central nervous system signs that were characterized by ataxia, recumbency and opisthotonus **(** **Fig. 2E** **)**. Macroscopic lesions typical of *S. suis* infection were identified in all animals infected with the WT strain. The predominant gross lesions observed in the WT-infected group were meningitis, lung lesions which were characterized by well-demarcated red-purple consolidation involving 10% to 20% of the lung, fibrin deposits in the abdominal cavity, and increased fluid in the joints. Obvious histopathological lesions were also observed **(Fig. S1)**. In comparison, animals inoculated with the Δ*hp0197* strain showed no clinical signs of infection, and all of the pigs survived for the duration of the test **(** **Fig. 2D** **)**. Similarly, no obvious macroscopic lesions or histopathological lesions associated with *S. suis* were observed in any animals in this group **(Fig. S1)**. *S. suis* was detected in the blood of the pigs in the WT group (range from 10^6^ to 10^8^ CFU ml^−1^), but no bacteria could be isolated from the blood of any of the pigs in the Δ*hp0197* group at any given point during the test. The experimental infection on pigs also indicated that *hp0197* contributed significantly to the virulence of SS2.

### Contribution of *hp0197* to the Resistance to Phagocytotic Killing and to CPS Synthesis

**Figure 3:**
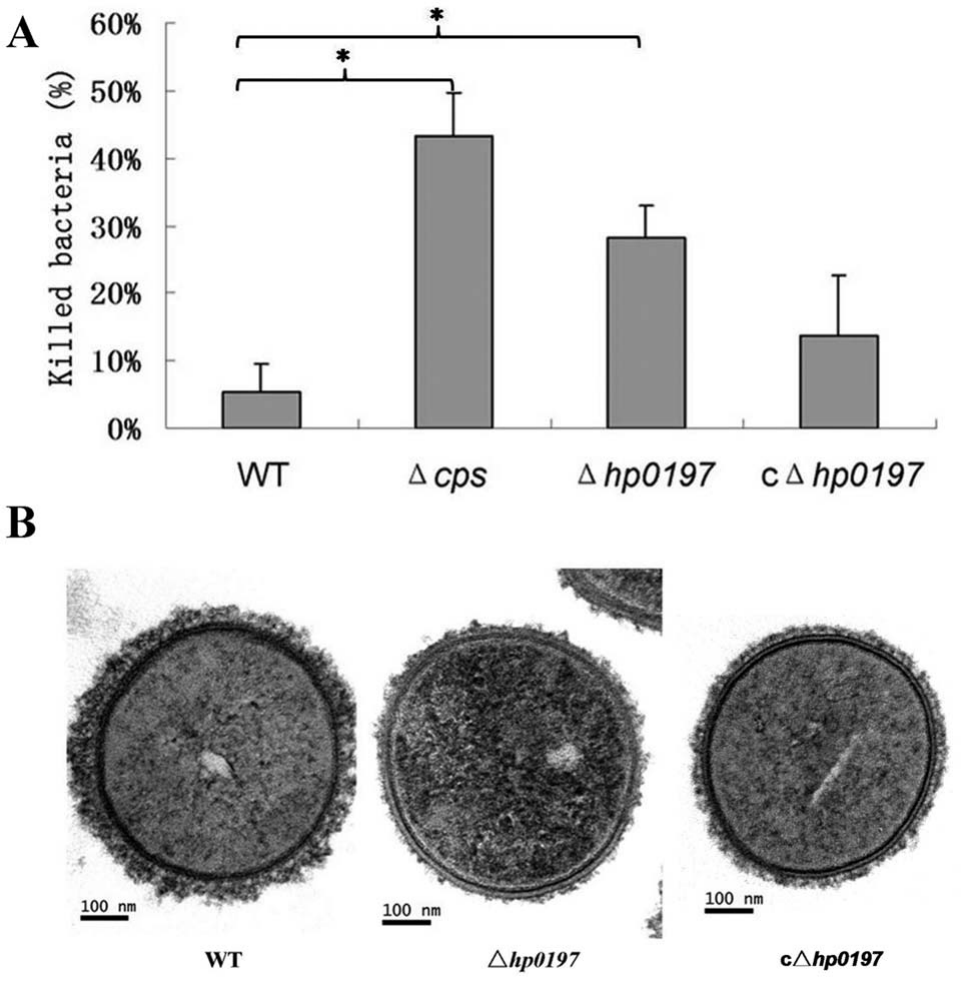
Contribution of *hp0197* to the resistance to phagocytotic killing and to CPS synthesis. (**A**) Percent of bacteria killed after 90 min incubation with murine phagocytes. The experiment was repeated five times. Error bars indicate standard deviations. “*” represents P value <0.05 of WT versus Δ*cps* group *or* WT versus Δ*hp0197* group *respectively.* (**B**) Morphology of the WT (left), the Δ*hp0197* (middle) and cΔ*hp0197* (right) strains. Transmission electron microscopy revealed that the WT strain was surrounded by a thick polysaccharide capsule (52.8±10.4 nm); the thickness of the Δ*hp0197* capsule was markedly reduced (1.1±0.3 nm), and cΔ*hp0197* could if not completely, partially restore this phenotype (21.1±6.6 nm).

The experimental infection demonstrated that Δ*hp0197* could be cleared easily during infection **(** **Fig. 2** **)**, which suggested that Δ*hp0197* was not as efficient in avoiding destruction by phagocytotic processes as its parent strain. To confirm this hypothesis, *in vitro* killing of the WT strain and Δ*hp0197* by phagocytes was compared in the presence of normal complete serum without opsonizing antibodies. Compared to the WT strain (only 5.0±4.2% of bacteria were killed), which could resist the bactericidal effect of phagocytes [18], [53], the isogenic *cps* mutant derived from strain 05ZYS (Δ*cps*) was readily destroyed by phagocytes as described before [18]. Interestingly, a notable number of the Δ*hp0197* bacteria (28.0±4.6%) were killed and the reintroduction of *hp0197* into Δ*hp0197* could recover the resistance to the phagocytic processes to some extent (14.1±9.1%) **(** **Fig. 3A** **).** Furthermore, these bacteria showed no significant difference without murine phagocytes. Therefore, the data suggested that *hp0197* directly or indirectly contributes to the resistance of *S. suis* to phagocytic killing.

CPS is thought to be a critical antiphagocytic factor that can down-regulate signaling pathways involved in macrophage phagocytosis, and could protect *S. suis* against killing and phagocytosis [54], [55]. The decreased capacity of Δ*hp0197* to resist phagocytotic killing encouraged us to test the effect of *hp0197* on CPS thickness. Electron microscopical characterization of the WT strain revealed a thick CPS, (52.8±10.4 nm). In contrast, Δ*hp0197* exhibited a markedly reduced thickness of the capsule (1.1±0.3 nm), corresponding to 2.1% of WT, and cΔ*hp0197* could restore the phenotype to some extent (21.1±6.6 nm) **(** **Fig. 3B** **)**. This suggested that *hp0197* could contribute to CPS thickness, which in turn affect the resistance of the bacteria to phagocytotic killing.

### Microarrays-based Comparative Transcriptomics Analysis of Δ*hp0197* and WT Suggests the Correlation between *hp0197* and CcpA Activity

**Figure 4:**
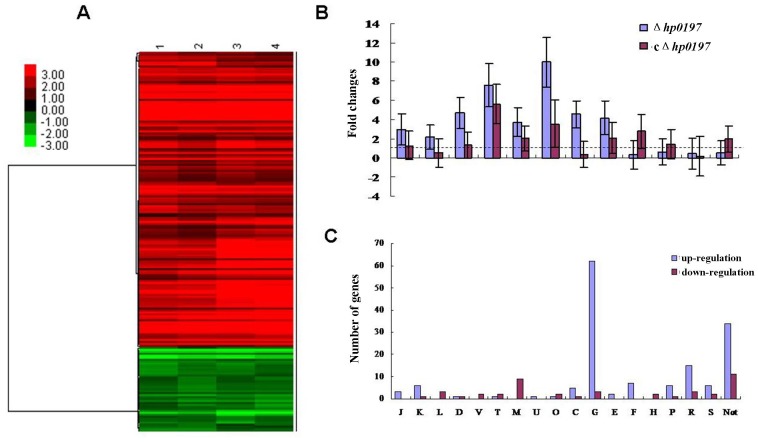
Microarray-based comparative transcriptomic analysis of Δ*hp0197* and WT strains. (**A**) Microarray-based comparative transcriptomic analysis of Δ*hp0197* and WT strains. A hierarchical cluster of 193 transcripts in the Δ*hp0197* compared to the WT strain. The increased and decreased transcript expression levels were indicated by red and green, respectively. (**B**) Different expression profiles identified by microarray analysis were validated by qRT-PCR based on the data obtained from a total of 12 genes. From left to right: ssu05_1372, ssu05_1933, ssu05_0167, ssu05_2076, ssu05_2137, ssu05_1039, ssu05_0360, ssu05_1401, ssu05_0812, ssu05_0573 (*cps 2J*), ssu05_0265 and ssu05_0469. (**C**) Functional classification of the differentially expressed genes. J: Translation, ribosomal structure and biogenesis; K: Transcription; L: Replication, recombination and repair; D: Cell cycle control and cell division; V: Defense mechanisms; T: Signal transduction mechanisms; M: Cell membrane and envelope biogenesis; U: Intracellular trafficking, secretion and vesicular transport; O: Posttranslational modification, protein turnover, chaperones; C: Energy production and conversion; G: Carbohydrate transport and metabolism; E: Amino acid transport and metabolism; F: Nucleotide transport and metabolism; H: Coenzyme transport and metabolism; I: Lipid transport and metabolism; P: Inorganic ion transport and metabolism; Q: Secondary metabolites biosynthesis, transport and catabolism; R: General function prediction only; S: Function unknown; Not: Not in COGs.

To gain insight into the role of *hp0197* in CPS synthesis and virulence, the transcriptome profile of Δ*hp0197* was compared to that of WT using a SS2 genomic microarray. A total of 193 genes were differentially expressed (change ratio> = 2, p-value <0.05), among which 150 genes were up-regulated and 43 genes were down-regulated **(** **Fig. 4A** **and Table S1, S2)**. The different expression patterns identified by the microarrays analysis were validated by qRT-PCR based on the data obtained from a total of 12 genes **(** **Fig. 4B** **)**, and the comparative proteomics based on the 2-DE/MS **(Fig. S2 and Table S3)** further partially confirmed the differential expression.

The functional classifications of these differentially expressed genes provided clear indications of the changes that occurred in Δ*hp0197* compared to WT **(** **Fig. 4C** **)**. It was intriguing that 48% (72 of 150) of the up-regulated genes were predicted to encode proteins involved in carbohydrate transport and metabolism (COG: G) when *hp0197* was absent, including ATP-binding cassette (ABC) transporters and PTS operons responsible for the transport of glucose, lactose, maltose/maltodextrin, mannose, fructose, and cellobiose **(Table S2)**.The differential expressions of these genes have also been documented in a study on Group A *Streptococcus* when *ccpa* (*catabolite control protein A*), a global regulator of sugar metabolism, was absent [56]. Among the up-regulated genes, 57% (86 of 150) were documented in the Δ*ccpa* originated from SS2 [57]. Not surprisingly, 8/19 genes loci which had a putative CcpA-binding sequence “*cre*” were significantly up-regulated. Interestingly, 39% (17 of 43) of the down-regulated genes showed lower expression levels in the isogenic *ccpa* mutant [57], including the entire operon locus responsible for CPS synthesis (COG: M), which was confirmed by qRT-PCR for *ssu05_0573* (*cps 2J*) (Fig. 4B). Additionally, lower expression of *hp0197* (*ssu98_0197* in strain 98HAH33 or *ssu05_0196* in strain 05ZYH33) was also observed in the Δ*ccpa* [57]. Therefore, a correlation must exist between *hp0197* and CcpA activity.

### *hp0197* Controls the CcpA Activity via HPr-Ser-46P

CcpA is a critical global regulator of carbohydrate metabolism and controls the expression of genes involved in complex carbohydrate utilization and virulence, such as an operon for CPS synthesis. A lower CcpA activity could contribute to the reduced thickness of the CPS and the decreased virulence in *group A Streptococcus* and *S. suis* [56], [57]. However, CcpA expression was not down-regulated as anticipation but up-regulated in Δ*hp0197*. Therefore, we attempted to determine why CcpA was up-regulated while the *cre*-binding activity was decreased in Δ*hp0197* mutant.

**Figure 5:**
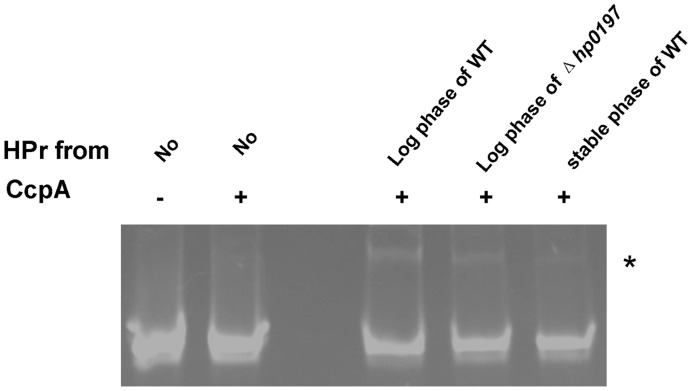
*hp0197* controls CcpA activity by HPr-Ser-46P. The purified FLAG-tagged HPr from the log-phase WT or Δ*hp0197* bacteria were compared to evaluate the binding of CcpA to its target site (*cre*) by EMSA. The HPr from the stable-phase WT strain served as a control. Significant more DNA (indicated by *) with CcpA and HPr from Log-phase WT migrated slower than DNA with CcpA and HPr from Δhp0197 or from the stable-phase WT strain, indicating that HPr from log-phase WT could enhance the binding of CcpA to *cre* and the HPr from log-phase Δ*hp0197*could not enhance the ability significantly.

CcpA activity is controlled by a complex interaction with HPr [58]. When glucose or other readily metabolized carbohydrates are present at sufficiently high concentrations, phosphorylation of the phosphocarrier protein HPr at residue Ser-46 (HPr-Ser-46P) occurs. This protein is a co-effector for CcpA that could mediate CcpA binding to *cre* sites [59]. To determine whether *hp0197* controls CcpA activity through HPr, we purified FLAG-tagged HPr from both the WT and Δ*hp0197* bacterial strains. Purified HPr from log-phase WT bacteria had good binding activity to *cre* in associated with purified recombinant His-tagged CcpA, but HPr from Δ*hp0197* had a weaker binding effect **(** **Fig. 5** **)**, indicating that lower levels of HPr-Ser-46-P were produced in Δ*hp0197*. Lower activity could also be observed when HPr was isolated from stable-phase WT bacteria, which had very low level of HPr-Ser-46 due to insufficient energy. These data supported the hypothesis that *hp0197* controlled CcpA activity by HPr-Ser-46P binding.

## Discussion

The 2005 Sichuan outbreak of *S. suis* provoked strong interest and extensive research; some progress has been achieved through genomic and proteomic studies [17], [60]. However, many aspects of the pathogenesis of the bacteria remain uncertain. For example, the biological properties of a few hypothetical proteins encoded by the bacteria need to be characterized.

Several hypothetical proteins are expressed on the surface of the *S. suis*, and several studies have suggested their potential roles in bacterial pathogenesis [47], [48], [50], [51], [61], [62]. HP0197 is one such hypothetical protein that does not have significant sequence homology to any other known protein and does not have similar domain architecture besides the G5 domain, which accounts for only 14% of the total amino acid length. These findings indicate that HP0197 is a novel protein. Furthermore, a previous study [51] demonstrated that HP0197 was an important vaccine candidate and was present in almost all tested pathogenic clinical SS2 isolates. Therefore, it was believed that clarifying its biological function would contribute to an increased understanding of its gene structure and its biological functions.

To clarify the pathogenic role of *hp0197* in *S. suis* virulence, mice and pigs were experimentally infected with the bacteria. WT strain cause high mortality in mice (80%) and pigs (100%), and mortality was significantly reduced when *hp0197* was absent; only 2 mice (n = 10) and no pig (n = 8) died during the experimental infection **(** **Fig. 2** **)**. Additionally, obvious clinical signs, significant pathological lesions, and high levels of bacteria in the blood were observed in the WT-infected animals. However, only slight or no significant clinical signs and pathological lesions were observed in mice or pigs inoculated with Δ*hp0197*, respectively **(Fig. S1)**. In contrast to pathogenic SS2, Δ*hp0197* infection did not induce high levels of cytokine expression [63], which could be provoked by highly pathogenic SS2 [55], [63], [64]. These data indicated that *hp0197* significantly contributed to *S. suis* virulence.

During the experimental infection, no bacteria could be isolated from the Δ*hp0197*-infected pigs, and the number of strains recovered from the blood in Δ*hp0197*-infected mice was much lower and was cleared quickly compared to WT **(** **Fig. 2C** **)**. These results suggested that Δ*hp0197* could not resist phagocytotic killing. The *in vitro* assay confirmed that *hp0197* played an essential role in the resistance of *S. suis* to phagocytic killing **(** **Fig. 3A** **)**; this was attributed to a reduced thickness of the CPS in Δ*hp0197* mutant **(** **Fig. 3B** **)**.

How could a surface protein control CPS thickness and also influence *S. suis* virulence? To answer this question, we compared the transcriptome profiles of Δ*hp0197* and WT, and determined that 53% (103 of 193) of the differentially expressed genes in Δ*hp0197* showed expression profiles that were similar to the Δ*ccpa* [57]. CcpA is a critical global regulator of carbohydrate metabolism and controls the expression of genes involved in complex carbohydrate utilization and virulence, such as an operon for CPS synthesis [56], [57]. Therefore, a correlation should exist between *hp0197* and CcpA activity. However, CcpA expression was not down-regulated but up-regulated in Δ*hp0197* **(Table S2)**. In fact, CcpA activity is controlled by a complex interaction with HPr [58]. When glucose or other readily metabolized carbohydrates are present at sufficiently high concentrations, HPr is phosphorylated at residue Ser-46 (HPr-Ser-46P), which subsequently served as a co-effector for CcpA that could mediates CcpA binding to *cre* sites [59]. Purified HPr from Δ*hp0197* had a decreased binding activity to CcpA compared to WT **(** **Fig. 5** **)**, suggesting that lower levels of HPr-Ser-46-P were produced in Δ*hp0197*. This result suggests that HP0197 contributed to the utilization of sugars. However, none of the genes responsible for glycolysis or for the phosphoenolpyruvate-dependent carbohydrate:phosphotransferase system (PTS) of glucose were observed to be down-regulated **(Table S1)** and some of them were even up-regulated **(Table S2)** in Δ*hp0197*. This suggested that HP0197 could not contribute to transport and glycolysis of glucose and might facilitates the uptake of saccharides.

**Figure 6:**
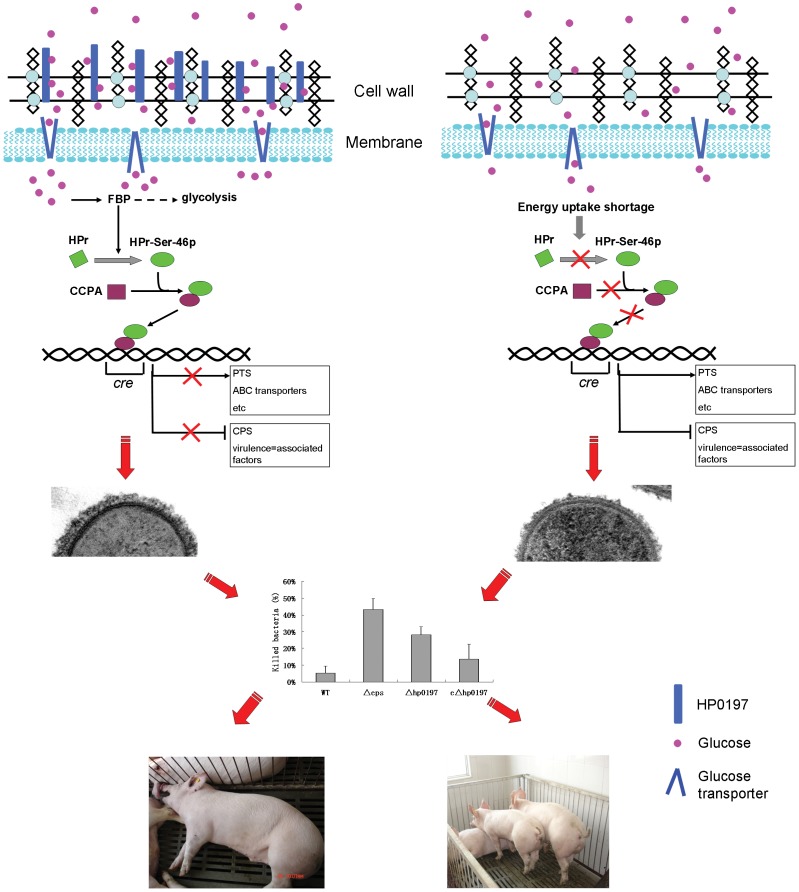
Schematic representation how HP0197 contributes the CPS sysnthesis and virulence of *Streptococcus suis* via CcpA activity. HP0197 could promote the level of phosphorylation of the phosphocarrier protein HPr at residue Ser-46 (HPr-Ser-46P), which could enhance the binding of CcpA to the *cre* of the target operons, subsequently control the expression of genes involved in carbohydrate utilization and CPS synthesis. A thin CPS could allow the pathogenic bacteria to be killed more easily by phagocytes and cleared from the host. Thus, HP0197 ultimately contributes to CPS synthesis and the virulence of *S. suis* via CcpA activity.

In summary, the deletion of *hp0197* would decrease the activity of CcpA, and would further alleviate the carbon catabolite repression and down-regulate genes for CPS synthesis. This would decrease the bacterial resistance to phagocytotic killing and would thus decrease the virulence of *S. suis* **(** **Fig. 6** **)**. In conclusion, HP0197 contributes to CPS synthesis and the virulence of *Streptococcus suis* via CcpA.

## Materials and Methods

### Bacterial Strains, Plasmids and Growth Conditions

*S. suis* serotype 2 strain 05ZY was chose as the WT strain. This strain was isolated from the brain of a diseased piglet and expresses muramidase-released protein, extracellular protein factor and suilysin was chosen as wild type (WT) strain [5]. A temperature-sensitive *S. suis*-*E. coli* shuttle vector (pSET4s), that carries spectinomycin resistance gene (*spc^r^*), was used to construct the Δ*hp0197* mutant. pSET2, a *S. suis-E. coli* shuttle vector, carrying *spc^r^*, was used in the construction of the complementary bacterium.

*S. suis* strains were grown in Todd–Hewitt broth (THB) or on Todd-Hewitt agar (THA) (Becton Dickinson, Sparks, MD, USA) at 37°C under aerobic conditions. *E. coli* (DH5α) was cultured in Luria-Bertani broth or agar (Becton Dickinson) at 37°C for 8 h. When necessary, spectinomycin was added to the culture media at the following concentrations: 50 µg/ml for *E. coli* and 100 µg/ml for *S. suis*.

### Construction of Δ*hp0197* and Complementation

The isogenic *hp0197* mutant was derived from the SS2 strain 05ZY using the strategy of allelic replacement mutagenesis. Approximately 600 bp of *hp0197* flanking sequence was obtained by PCR using the 05ZY chromosome as a template with the primer pairs HP0197L1/HP0197L2 (left arm) or HP0197R1/HP0197R2 (right arm). An *egfp*-conding region was amplified from plasmid pEGFP-N1 with primer pairs EGFP-1/EGFP-2 to exchange *egfp* with the *hp0197* coding sequence **(Table S4)**. All PCR amplicons were digested with the appropriate restriction enzymes and were sequentially ligated into the temperature-sensitive *S. suis*-*E. coli* shuttle vector pSET4s **(** **Fig. 1** **)** for constructing the knockout vector pSET4s::*hp0197*. To obtain the isogenic *hp0197* mutant, competent cells of *S. suis* were subjected to electrotransformation with pSET4s::*hp0197* and were grown at 28°C in the presence of spectinomycin selection as described by Takamatsu *et al.* [65]. Bacteria at the mid-logarithmic growth phase were diluted with THB containing spectinomycin and cultured at 28°C to early logarithmic phase. The culture was then shifted to 37°C and incubated for 4 h. Subsequently, the cells were spread on THA and incubated at 28°C. Temperature resistant colonies were screened at 37°C for the loss of vector-mediated spectinomycin resistance, and the putative mutants were detected in which the *hp0197* gene was replaced by e*gfp* gene as a consequence of homologous recombination via a double cross-over. The deletion of *hp0197* in SS2 was further confirmed by PCR analysis with the HP0197-P1/HP0197-P2 and EGFP-L/EGFP-R primer pairs, and by immunoblotting with antiserum raised against purified recombinant HP0197 protein [51].

To compliment *hp0197*, a DNA fragment containing the *hp0197* gene and its predicted upstream promoter was amplified by PCR with primers cHP0197-1/cHP0197-2 **(Table S4)**. The obtained DNA fragment was digested with the appropriate restriction enzymes and was cloned into the *E. coli*-*S. suis* shuttle vector pSET2 to generate the recombinant plasmid pSET2::C*hp0197*. The plasmid was then introduced into Δ*hp0197* to screen the complemented cΔ*hp0197* strain on THA with selection pressure of spectinomycin. The HP0197 expression was confirmed by the immunoblotting with the antiserum as previously described.

### Experimental Infections of Mice and Pigs

This study was performed in strict accordance with the Guide for the Care and Use of Laboratory Animals Monitoring Committee of Hubei Province, China, and the protocol was approved by the Committee on the Ethics of Animal Experiments at the College of Veterinary Medicine, Huazhong Agricultural University (Permit Number: 00010399). All efforts were made to minimize the suffering of the animals.

Thirty 6-week-old female CD-1 mice were randomly assigned to 3 groups with 10 mice in each group. Group 1 was inoculated by intraperitoneal injection of 1 ml of a WT strain suspension at 10^9^ CFU/ml, and groups 2 and 3 received the same dose of the Δ*hp0197* and cΔ*hp0197*, respectively. The mice were monitored constantly three times a day for 7 days for clinical signs, and were assigned clinical scores as described by Dominguez-Punaro [66]. Mice exhibiting extreme lethargy or neurological signs were considered moribund and were humanely euthanized. The survived animals were sacrificed via carbon dioxide inhalation at the end of the experiment. To evaluate the bacterial load, blood samples were collected daily from the tail vein and at the time of euthanasia by cardiac puncture and were plated on THA plates.

A total of 20 healthy pigs (ages 4–5 weeks) from a herd that was free of SS2 were assigned to three groups. One hour before inoculation, all pigs were given 2 ml of 2% acetic acid intranasally to enhance the severity of the *S. suis* challenge [67]. Pigs in group 1 (n = 8) and group 2 (n = 8) were inoculated intranasally with 2 ml of 10^7^ CFU of WT and Δ*hp0197* strains, respectively. Group 3 (n = 4) was inoculated with PBS as a control. Clinical signs of infection and the presence of *S. suis* in the blood were monitored during the trial. Clinical symptoms were quantified by clinical scores as described by Li *et al* [68]. Briefly, a daily clinical score (from 0 to 8) was derived as the sum of the attitude and locomotion scores for each pig. The scores were based upon signs of nervous, musculoskeletal, and respiratory diseases. Attitude scores were given as follows: 0 = normal attitude and response to stimuli; 1 = inactive and slow to respond, with oculonasal secretions; 2 = only responsive to repeated stimuli; 3 = recumbent, nonresponsive, and unaware of surroundings; and 4 = dead. Locomotion scores were given as follows: 0 = normal gait and posture; 1 = slight lack of coordination, lameness, and/or joint swelling but able to stand without assistance; 2 = clearly uncoordinated or lame but able to stand without assistance; 3 = severe lameness and/or severe ataxia; and 4 = dead. Pigs with a clinical score of >2 on either scale were euthanized by lethal injection. The survived animals in all three test groups were sacrificed at day 7 pi and were examined for pathological lesions.

### Killing by Murine Phagocytes

Murine phagocytes were isolated as previously described [69]. Blood samples were collected by venous puncture from mice, and cell populations were separated by Ficoll 400-Hypaque density gradient centrifugation. The murine phagocytes were isolated by sedimentation in 6% dextran and the contaminating erythrocytes were removed by lysis with 0.83% ammonium chloride. The collected phagocytes were counted by trypan blue staining, and then were adjusted to a density of 1×10^6^ cells/ml. For each sample, 100 µl of bacteria at a concentration of approximately 1×10^4^ CFU/ml was opsonized with an equal volume of complete normal murine serum for 30 min at 37°C and was mixed in a microcentrifuge tube with 100 µl of murine phagocytes. The mixture was incubated for 90 min at 37°C in a 5% CO_2_ environment. The bacteria were not toxic to the phagocyte under these conditions [18]. After incubation, the cells were lysed with sterile water. Viable bacterial counts were determined by plating the bacteria onto THA. The parallel experiment without phagocytes has been done to evaluate the killing effects of serum.

### Electron Microscopy

For morphological analysis of the capsule structure, samples from early exponential grown bacteria were fixed according to a lysine/acetate-based formaldehyde/glutaraldehyde ruthenium red-osmium fixation procedure as previously described [70]. In addition, the capsule thickness was measured from randomly selected bacteria using the iTEM software.

### Microarray-based Comparative Transcriptomic Analysis of Δ*hp0197*

Based on the whole genome sequence of 05ZYH33, specific 40- to 60-mer oligo-nucleotides were designed to cover every putative ORF (2194 in total) in the genome using the eArray EArray Online Microarray Design Tool (https://earray.chem.agilent.com/earray/) and were printed six times on the surface of each microarray slide (Agilent).

Total RNAs was extracted with Trizol reagent (Invitrogen) from Δ*hp0197* or WT strain cultures grown in THB to an OD_600_ of 0.35, and were purified using an RNeasy Mini Kit (Qiagen). RNA was reverse-transcribed and was then transcribed into cRNA. Purified cRNA from Δ*hp0197* or WT strain were labeled with Cy5 NHS ester or Cy3 NHS ester (GE healthcare), respectively and were purified again. After fragmentation, microarray slides were hybridized with 1 µg of Cy5- or Cy3-labeled cDNAs at 65°C for 17 h with 10 rpm rotation. All hybridization slides were scanned by an Agilent Microarray Scanner System (G2565BA) after appropriate washing. The p-values and corrected p-values was calculated by Benjamin-Hochberg FDR estimation (GeneSpring software 11.0, Agilent) in the four independent bio-replicates. Only those genes with more than twice of change ratio and corrected a p-value <0.05 were selected as differentially expressed genes.

### qRT-PCR Evaluation

To validate the data from the microarray analysis, 12 genes and the *hp0197* flanking genes (SSU98_0196 and SSU98_0198) **(Table S5)** were selected to evaluate the expression changes in WT and Δ*hp0197* by qRT-PCR with SYBR Green detection [71]. Three independent cultures of each strain (WT and Δ*hp0197*) grown in THB to an OD_600_ of 0.35 were sampled and quenched in liquid nitrogen. Total RNAs were extracted with Trizol reagent (Invitrogen) and were purified using an RNeasy Mini Kit (Qiagen). The quality and quantity were determined by a 2100 Agilent bioanalyzer and by Nanodrop ND1000 UV spectroscopy. Quantitative analysis was performed in triplicate with an ABI PRISM 7500 sequence detection system using RNA from three independent cultures of each strain (WT and Δ*hp0197*). 16SrRNA was chosen as the internal control. Various Ct values were normalized to the average Ct value for 16SrRNA. The mean fold changes in target gene expression was calculated as described by Livak & Schmittgen [72].

### Determination of CcpA Activity by Electrophoretic Mobility Shift Assay (EMSA)

The purified FLAG-tagged HPr from log-phase and stable-phase of WT and Δ*hp0197* were compared to determine the binding of CcpA to *cre* as described by Lance M Hellman and Michael G Fried [73]. Purified recombinant His-tagged CcpA (1.5 µg), along with 1.5 µg of purified HPr and 0.3 µg of DNA harboring *cre*, were prepared and subjected to electrophoresis under native conditions in a polyacrylamide gel. After electrophoresis, the distribution of species containing nucleic acid was determined by EB staining.

### Statistical Analysis

Unless otherwise specified, data were analyzed by two-tailed, unpaired t-tests, and all assays were repeated at least three times. The data were expressed the means ± standard deviations. For *in vivo* virulence experiments, survival was analyzed with the Log-Rank test. For all tests, a value of P<0.05 was considered as the threshold for significance.

## Supporting Information

Figure S1 **Relevant histopathological findings could be observed from WT-infected pigs (day 3 pi) while no significant histopathological findings could be observed from a representative pig from the Δ** ***hp0197*** **group (day 7 pi).** From a pig in the WT group, moderate inflammatory cells were observed in the inner layer of the dura mater (A), along with leptomeningitis with intense inflammation consisting of neutrophils and macrophages (C). No significant findings were observed from the pigs in the Δ*hp0197* mutant group (B and D). Scale bar = 100 µm.
(JPG)

Figure S2 **2-DE map of whole cellular proteins from the WT (A) and Δ** ***hp0197*** **(B) strains.** Sixteen spots corresponding to 9 proteins were found differentially expressed **(Table S3)**.
(TIF)

Table S1 **Reduced expression levels of genes in Δ** ***hp0197*** **compared to WT confirmed by microarray analysis.**
(DOC)

Table S2 **Increased expression levels of genes in Δ** ***hp0197*** **compared to WT confirmed by microarrays analysis.**
(DOC)

Table S3 **Differential expression levels of proteins in Δ** ***hp0197*** **compared to WT as identified by 2-DE/MS.**
(DOC)

Table S4 **Primer sequences used for this study.**
(DOC)

Table S5 **Primer sequences used for qRT-PCR.**
(DOC)
