# Supplementary figures and images for: HP0197 Contributes to CPS Synthesis and the Virulence of Streptococcus suis via CcpA
Source: PLoS One. 2012 Nov 30;7(11):e50987. doi: 10.1371/journal.pone.0050987 (PMC3511442; doi:10.1371/journal.pone.0050987)

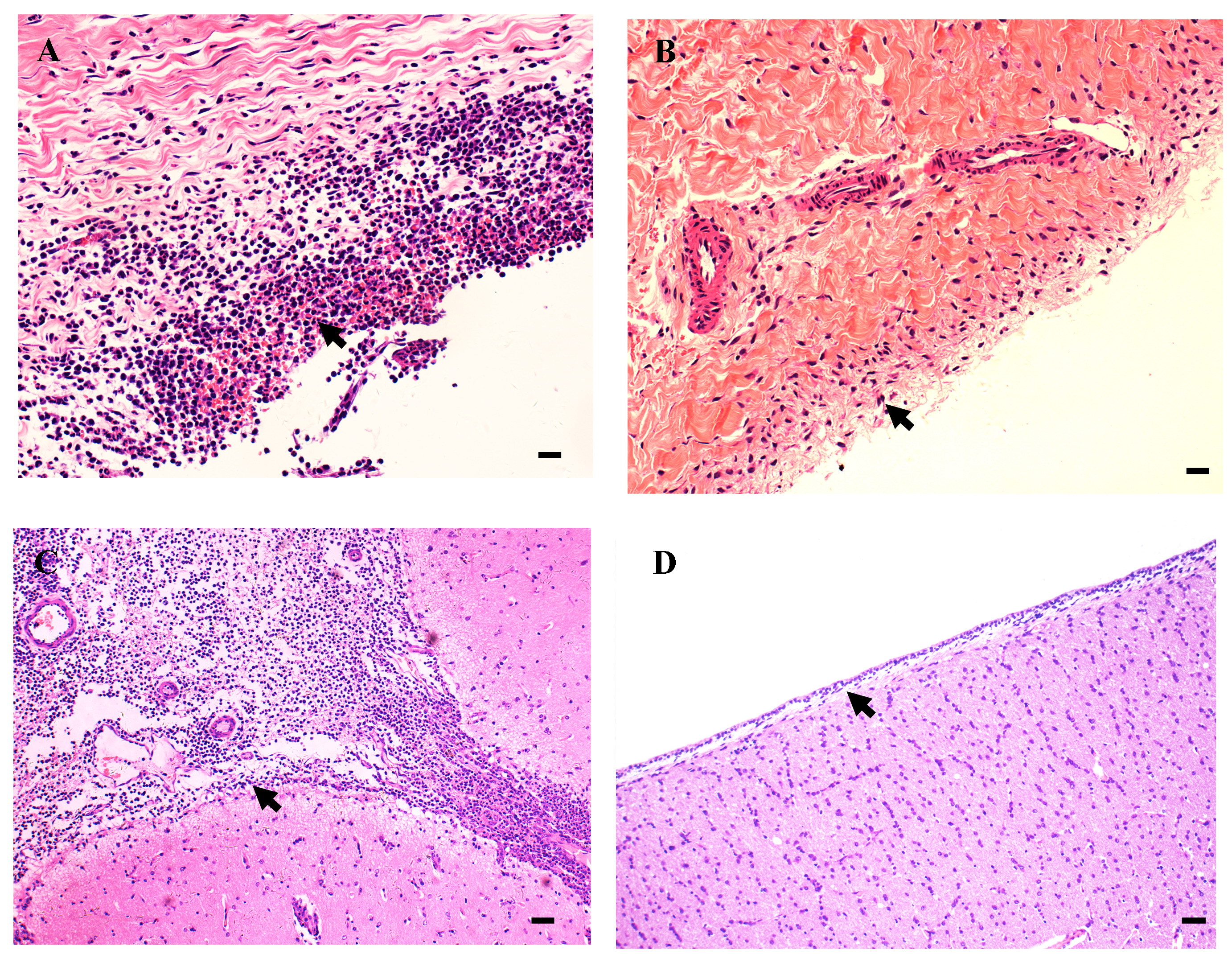

Supplement: Figure S1 — Relevant histopathological findings could be observed from WT-infected pigs (day 3 pi) while no significant histopathological findings could be observed from a representative pig from the Δ hp0197 group (day 7 pi). From a pig in the WT group, moderate inflammatory cells were observed in the inner layer of the dura mater (A), along with leptomeningitis with intense inflammation consisting of neutrophils and macrophages (C). No significant findings were observed from the pigs in the Δhp0197 mutant group (B and D). Scale bar = 100 µm. (JPG) [file pone.0050987.s001.jpg]

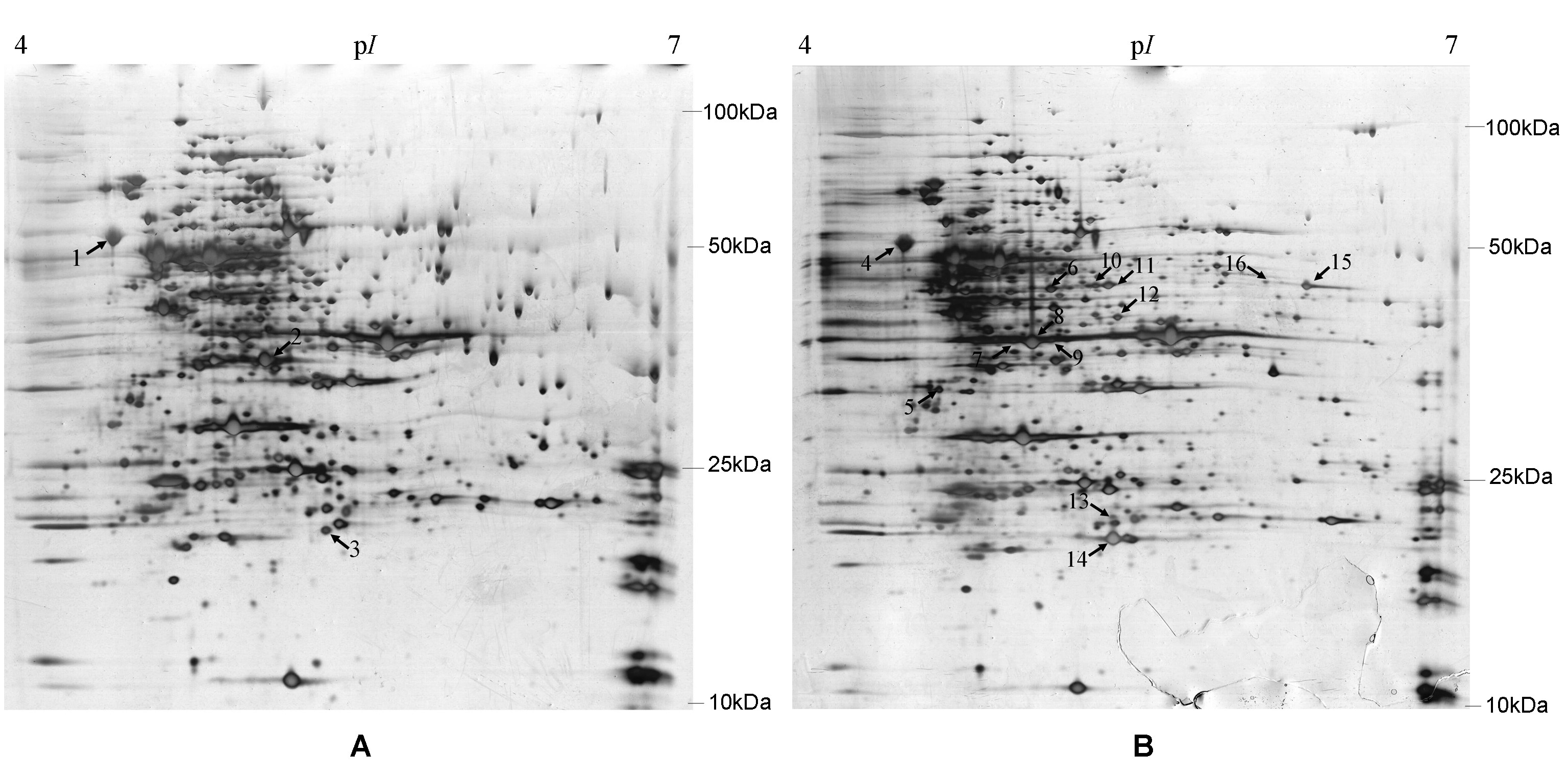

Supplement: Figure S2 — 2-DE map of whole cellular proteins from the WT (A) and Δ hp0197 (B) strains. Sixteen spots corresponding to 9 proteins were found differentially expressed (Table S3). (TIF) [file pone.0050987.s002.tif]
