# Supplementary material for: HP0197 Contributes to CPS Synthesis and the Virulence of Streptococcus suis via CcpA
Source: PLoS One. 2012 Nov 30;7(11):e50987. doi: 10.1371/journal.pone.0050987 (PMC3511442; doi:10.1371/journal.pone.0050987)
Supplement: Table S1 — Reduced expression levels of genes in Δ hp0197 compared to WT confirmed by microarray analysis. (DOC) [file pone.0050987.s003.doc]

**Table S1. Reduced expression levels of genes in Δ*hp0197* compared to WT confirmed by microarray analysis**

| Code for ORF | Fold changes  (WT/Δ*salp*) | Corrected  p-value | Functional annotation | *Genes in (Δ*ccpa*) |
| --- | --- | --- | --- | --- |
| SSU05_0114 | 2.1638 | 0.0297 | hypothetical protein SSU05_0114 |  |
| SSU05_0125 | 2.0868 | 0.0429 | hypothetical protein SSU05_0125 |  |
| SSU05_0180 | 2.4719 | 0.0084 | hypothetical protein SSU05_0180 |  |
| SSU05_0181 | 3.8435 | 0.0312 | ABC-type multidrug transport system, ATPase component |  |
| SSU05_0196 | 262.3685 | 0.0015 | hypothetical protein SSU05_0196 | + |
| SSU05_0265 | 5.8709 | 0.0025 | putative effector of murein hydrolase LrgA |  |
| SSU05_0266 | 6.3720 | 0.0072 | putative effector of murein hydrolase |  |
| SSU05_0267 | 6.3330 | 0.0084 | putative effector of murein hydrolase |  |
| SSU05_0268 | 7.4182 | 0.0291 | hypothetical protein SSU05_0268 | + |
| SSU05_0429 | 2.0513 | 0.0445 | hypothetical protein SSU05_0429 |  |
| SSU05_0430 | 2.4826 | 0.0175 | Signal transduction histidine kinase |  |
| SSU05_0431 | 2.0746 | 0.0170 | hypothetical protein SSU05_0431 |  |
| SSU05_0468 | 2.9084 | 0.0212 | membrane GTPase involved in stress response | + |
| SSU05_0469 | 2.5848 | 0.0185 | hypothetical protein SSU05_0469 |  |
| SSU05_0564 | 2.2136 | 0.0138 | Cps2A |  |
| SSU05_0565 | 2.0926 | 0.0292 | Cps2B | + |
| SSU05_0566 | 2.1506 | 0.0351 | Cps2C | + |
| SSU05_0567 | 2.6021 | 0.0084 | Cps2D | + |
| SSU05_0568 | 2.4341 | 0.0171 | Cps2E | + |
| SSU05_0569 | 2.3715 | 0.0158 | Cps2F | + |
| SSU05_0570 | 2.1730 | 0.0351 | glycosyltransferase | + |
| SSU05_0571 | 2.3269 | 0.0172 | Cps2H | + |
| SSU05_0573 | 2.4860 | 0.0228 | Cps2J | + |
| SSU05_0574 | 2.0268 | 0.0474 | cell wall biosynthesis glycosyltransferase | + |
| SSU05_0576 | 2.2440 | 0.0157 | hypothetical protein SSU05_0576 |  |
| SSU05_0578 | 2.3163 | 0.0314 | sialic acid synthase | + |
| SSU05_0687 | 2.3616 | 0.0426 | hypothetical protein SSU05_0687 |  |
| SSU05_0689 | 2.0048 | 0.0088 | phosphopantothenate--cysteine ligase |  |
| SSU05_0811 | 3.6340 | 0.0486 | subtilisin-like serine protease |  |
| SSU05_0812 | 2.8524 | 0.0318 | subtilisin-like serine protease |  |
| SSU05_1076 | 3.3342 | 0.0101 | L-lactate dehydrogenase | + |
| SSU05_1129 | 3.2781 | 0.0163 | hypothetical protein SSU05_1129 | + |
| SSU05_1197 | 2.1857 | 0.0350 | lantibiotic efflux protein |  |
| SSU05_1348 | 2.5870 | 0.0224 | cation transport ATPase | + |
| SSU05_1423 | 2.5460 | 0.0175 | transposase |  |
| SSU05_1424 | 2.6095 | 0.0292 | transposase |  |
| SSU05_1425 | 2.5259 | 0.0277 | transposase |  |
| SSU05_1638 | 2.3647 | 0.0393 | phosphoglycerate mutase 1 | + |
| SSU05_1639 | 2.6112 | 0.0448 | hypothetical protein SSU05_1639 |  |
| SSU05_1788 | 2.7022 | 0.0291 | hypothetical protein SSU05_1788 |  |
| SSU05_1892 | 2.2321 | 0.0277 | dihydroxy-acid dehydratase |  |
| SSU05_1932 | 2.7703 | 0.0074 | glucokinase regulatory protein |  |

*In this column, “+” indicated that the genes with reduced expression levels in Δ*hp0197* were also down-regulated in Δ*ccpa.*
