# Supplementary material for: HP0197 Contributes to CPS Synthesis and the Virulence of Streptococcus suis via CcpA
Source: PLoS One. 2012 Nov 30;7(11):e50987. doi: 10.1371/journal.pone.0050987 (PMC3511442; doi:10.1371/journal.pone.0050987)
Supplement: Table S2 — Increased expression levels of genes in Δ hp0197 compared to WT confirmed by microarrays analysis. (DOC) [file pone.0050987.s004.doc]

**Table S2. Increased expression levels of genes in Δ*hp0197* compared to WT confirmed by microarrays analysis**

| Code for ORF | Fold changes (Δ*salp*/WT) | Corrected p-value | Functional annotation | *Genes in (*Δccpa*) |
| --- | --- | --- | --- | --- |
| SSU05_0135 | 2.4571 | 0.0306 | acetate kinase | + |
| SSU05_0167 | 3.2192 | 0.0197 | transcriptional regulator | + |
| SSU05_0168 | 42.4073 | 0.0004 | ABC-type sugar transport system, periplasmic component | + |
| SSU05_0169 | 37.3662 | 0.0004 | ABC-type sugar transport system, periplasmic component |  |
| SSU05_0170 | 39.7089 | 0.0036 | ABC-type sugar transport system, permease component | + |
| SSU05_0171 | 38.7713 | 0.0068 | ABC-type sugar transport system, permease component | + |
| SSU05_0172 | 23.4181 | 0.0055 | Alpha-galactosidase | + |
| SSU05_0173 | 36.4825 | 0.0055 | hypothetical protein SSU05_0173 |  |
| SSU05_0175 | 2.1152 | 0.0224 | hypothetical protein SSU05_0175 |  |
| SSU05_0176 | 2.2105 | 0.0175 | hypothetical protein SSU05_0176 |  |
| SSU05_0212 | 4.0106 | 0.0314 | phosphotransferase system cellobiose-specific component IIC |  |
| SSU05_0213 | 10.6478 | 0.0169 | hypothetical protein SSU05_0213 | + |
| SSU05_0214 | 4.9617 | 0.0289 | ABC-type xylose transport system, periplasmic component |  |
| SSU05_0215 | 4.9956 | 0.0186 | hypothetical protein SSU05_0215 | + |
| SSU05_0216 | 3.5855 | 0.0486 | putative ABC-transport protein, membrane component | + |
| SSU05_0217 | 3.0221 | 0.0486 | ABC-type nitrate/sulfonate/bicarbonate transport system, ATPase component |  |
| SSU05_0218 | 2.7660 | 0.0445 | ABC-type nitrate/sulfonate/bicarbonate transport system, periplasmic component |  |
| SSU05_0219 | 2.2662 | 0.0253 | 6-phosphogluconolactonase/glucosamine-6-phosphate isomerase/deaminase |  |
| SSU05_0230 | 5.7038 | 0.0377 | glycosidase | + |
| SSU05_0231 | 6.1637 | 0.0491 | phosphotransferase system IIC component, glucose/maltose/N-acetylglucosamine-specific | + |
| SSU05_0256 | 2.4268 | 0.0277 | ABC-type uncharacterized transport system, permease component |  |
| SSU05_0258 | 1.9364 | 0.0303 | ABC-type uncharacterized transport system, ATPase component |  |
| SSU05_0280 | 8.4837 | 0.0117 | bifunctional acetaldehyde-CoA/alcohol dehydrogenase | + |
| SSU05_0360 | 5.9591 | 0.0185 | galactokinase |  |
| SSU05_0361 | 5.8912 | 0.0297 | galactose-1-phosphate uridylyltransferase | + |
| SSU05_0382 | 6.6081 | 0.0036 | hypothetical protein SSU05_0382 | + |
| SSU05_0383 | 5.2672 | 0.0047 | putative ATP-binding protein | + |
| SSU05_0384 | 5.0832 | 0.0063 | hypothetical protein SSU05_0384 |  |
| SSU05_0385 | 4.9019 | 0.0025 | hypothetical protein SSU05_0385 | + |
| SSU05_0386 | 3.7260 | 0.0271 | hypothetical protein SSU05_0386 |  |
| SSU05_0388 | 4.9781 | 0.0036 | N-formylmethionyl-tRNA deformylase | + |
| SSU05_0396 | 52.1920 | 0.0293 | metal-dependent hydrolase |  |
| SSU05_0397 | 42.7616 | 0.0245 | phosphotransferase system IIC component, glucose/maltose/N-acetylglucosamine-specific |  |
| SSU05_0398 | 16.4029 | 0.0218 | phosphotransferase system IIC component, glucose/maltose/N-acetylglucosamine-specific |  |
| SSU05_0439 | 6.1952 | 0.0106 | ribosome-associated protein Y (PSrp-1) | + |
| SSU05_0450 | 9.0739 | 0.0337 | phosphotransferase system, mannose/fructose/N-acetylgalactosamine-specific component IIB |  |
| SSU05_0627 | 18.2943 | 0.0237 | carbamate kinase | + |
| SSU05_0628 | 40.9160 | 0.0378 | hypothetical protein SSU05_0628 | + |
| SSU05_0655 | 2.7807 | 0.0390 | hypothetical protein SSU05_0655 | + |
| SSU05_0686 | 3.6612 | 0.0189 | phosphomannomutase | + |
| SSU05_0721 | 4.0258 | 0.0409 | hypothetical protein SSU05_0721 |  |
| SSU05_0723 | 17.4145 | 0.0055 | alpha-glycerophosphate oxidase | + |
| SSU05_0730 | 3.2025 | 0.0357 | NAD(FAD)-dependent dehydrogenase | + |
| SSU05_0736 | 11.6849 | 0.0055 | hypothetical protein SSU05_0736 | + |
| SSU05_0737 | 8.4654 | 0.0101 | uridine phosphorylase | + |
| SSU05_0738 | 10.6778 | 0.0014 | uridine phosphorylase |  |
| SSU05_0739 | 11.1229 | 0.0017 | hypothetical protein SSU05_0739 | + |
| SSU05_0740 | 11.7569 | 0.0004 | cobalt ABC transporter permease protein | + |
| SSU05_0741 | 12.0659 | 0.0015 | cobalt ABC transporter ATP-binding protein | + |
| SSU05_0742 | 13.0301 | 0.0004 | cobalt ABC transporter ATP-binding protein | + |
| SSU05_0743 | 3.1765 | 0.0101 | HAD superfamily hydrolase | + |
| SSU05_0744 | 2.2784 | 0.0103 | hypothetical protein SSU05_0744 |  |
| SSU05_0822 | 7.2465 | 0.0327 | sugar metabolism transcriptional regulator | + |
| SSU05_0823 | 6.0161 | 0.0175 | fructose-1-phosphate kinase-like protein | + |
| SSU05_0824 | 7.6012 | 0.0224 | phosphotransferase system, fructose-specific IIC component | + |
| SSU05_0825 | 6.7287 | 0.0169 | phosphotransferase system, fructose-specific IIC component |  |
| SSU05_0845 | 2.6054 | 0.0049 | hypothetical protein SSU05_0845 | + |
| SSU05_0882 | 3.1156 | 0.0175 | phosphomannomutase | + |
| SSU05_0902 | 4.7675 | 0.0351 | HAD superfamily hydrolase | + |
| SSU05_0999 | 4.8715 | 0.0245 | nucleotide-binding protein implicated in inhibition of septum formation | + |
| SSU05_1013 | 4.9147 | 0.0179 | ADP-glucose pyrophosphorylase | + |
| SSU05_1014 | 6.7307 | 0.0175 | ADP-glucose pyrophosphorylase |  |
| SSU05_1015 | 7.2554 | 0.0171 | glycogen synthase | + |
| SSU05_1016 | 6.5480 | 0.0175 | 1,4-alpha-glucan branching enzyme | + |
| SSU05_1035 | 3.8851 | 0.0168 | galactose mutarotase-like protein |  |
| SSU05_1036 | 4.3854 | 0.0156 | 6-phospho-beta-galactosidase |  |
| SSU05_1037 | 4.3271 | 0.0084 | phosphotransferase system cellobiose-specific component IIC | + |
| SSU05_1038 | 4.0279 | 0.0175 | phosphotransferase system cellobiose-specific component IIA |  |
| SSU05_1039 | 4.0166 | 0.0185 | transcriptional antiterminator |  |
| SSU05_1079 | 2.3291 | 0.0348 | ABC transporter permease | + |
| SSU05_1080 | 2.6305 | 0.0293 | ABC-type uncharacterized transport system, permease component | + |
| SSU05_1081 | 2.0728 | 0.0291 | ABC-type uncharacterized transport system, ATPase component | + |
| SSU05_1083 | 2.5157 | 0.0267 | ABC transporter periplasmic protein | + |
| SSU05_1084 | 2.4849 | 0.0171 | cytidine deaminase | + |
| SSU05_1085 | 3.0085 | 0.0058 | deoxyribose-phosphate aldolase | + |
| SSU05_1086 | 4.4873 | 0.0079 | pyrimidine-nucleoside phosphorylase | + |
| SSU05_1087 | 3.6563 | 0.0435 | 16S RNA G1207 methylase RsmC | + |
| SSU05_1153 | 8.1727 | 0.0397 | Beta-glucosidase-related glycosidase | + |
| SSU05_1154 | 8.3105 | 0.0337 | hypothetical protein SSU05_1154 |  |
| SSU05_1155 | 5.4940 | 0.0357 | phosphatase | + |
| SSU05_1156 | 8.5532 | 0.0397 | D-mannonate oxidoreductase |  |
| SSU05_1158 | 7.6770 | 0.0381 | glucuronate isomerase |  |
| SSU05_1159 | 7.1052 | 0.0317 | glucuronate isomerase |  |
| SSU05_1202 | 2.1323 | 0.0317 | isocitrate dehydrogenase |  |
| SSU05_1204 | 2.3498 | 0.0175 | methylcitrate synthase |  |
| SSU05_1205 | 2.4267 | 0.0277 | aconitate hydratase |  |
| SSU05_1211 | 18.0957 | 0.0224 | hypothetical protein SSU05_1211 |  |
| SSU05_1212 | 19.0933 | 0.0156 | hyaluronidase |  |
| SSU05_1213 | 23.4202 | 0.0187 | hyaluronidase |  |
| SSU05_1214 | 23.0837 | 0.0185 | hyaluronidase |  |
| SSU05_1215 | 23.0847 | 0.0175 | hyaluronidase |  |
| SSU05_1216 | 15.8949 | 0.0163 | preprotein translocase subunit YajC | + |
| SSU05_1217 | 10.9221 | 0.0277 | phosphotransferase system, mannose/fructose/N-acetylgalactosamine-specific component IID | + |
| SSU05_1218 | 9.8740 | 0.0169 | phosphotransferase system, mannose/fructose/N-acetylgalactosamine-specific component IIC | + |
| SSU05_1219 | 14.4980 | 0.0224 | phosphotransferase system, mannose/fructose/N-acetylgalactosamine-specific component IIB | + |
| SSU05_1220 | 12.1578 | 0.0189 | hypothetical protein SSU05_1220 | + |
| SSU05_1221 | 4.9364 | 0.0160 | phosphotransferase system, mannose/fructose-specific component IIA | + |
| SSU05_1222 | 3.7273 | 0.0237 | keto-hydroxyglutarate-aldolase/keto-deoxy-phosphogluconate aldolase |  |
| SSU05_1223 | 3.3119 | 0.0101 | ribokinase family sugar kinase |  |
| SSU05_1224 | 3.6059 | 0.0140 | hypothetical protein SSU05_1224 |  |
| SSU05_1225 | 4.0468 | 0.0114 | gluconate 5-dehydrogenase |  |
| SSU05_1336 | 6.5070 | 0.0175 | Beta-fructosidases (levanase/invertase) |  |
| SSU05_1337 | 5.7873 | 0.0345 | Beta-fructosidases (levanase/invertase) | + |
| SSU05_1338 | 6.8709 | 0.0357 | ABC-type sugar transport system, periplasmic component | + |
| SSU05_1339 | 5.9639 | 0.0351 | ABC-type sugar transport system, permease component | + |
| SSU05_1340 | 6.7027 | 0.0245 | ABC-type polysaccharide transport system, permease component |  |
| SSU05_1372 | 2.7310 | 0.0395 | transcriptional regulator |  |
| SSU05_1387 | 9.4004 | 0.0385 | amylase-binding protein B | + |
| SSU05_1401 | 14.1419 | 0.0156 | phosphotransferase system IIC component, glucose/maltose/N-acetylglucosamine-specific |  |
| SSU05_1417 | 3.3805 | 0.0250 | hypothetical protein SSU05_1417 | + |
| SSU05_1443 | 3.9234 | 0.0055 | maltodextrin phosphorylase |  |
| SSU05_1444 | 4.0995 | 0.0088 | maltodextrin phosphorylase | + |
| SSU05_1445 | 2.4659 | 0.0271 | purine-nucleoside phosphorylase | + |
| SSU05_1446 | 2.1884 | 0.0179 | purine nucleoside phosphorylase | + |
| SSU05_1447 | 2.1367 | 0.0175 | arsenate reductase | + |
| SSU05_1448 | 2.2073 | 0.0271 | phosphopentomutase | + |
| SSU05_1555 | 2.4650 | 0.0228 | glycosidase |  |
| SSU05_1667 | 6.1496 | 0.0084 | pyruvate-formate lyase activating enzyme | + |
| SSU05_1778 | 4.3253 | 0.0156 | putative PTS system, mannose-specific component IIAB | + |
| SSU05_1779 | 4.4497 | 0.0185 | mannose-specific PTS IIC | + |
| SSU05_1780 | 4.8904 | 0.0047 | mannose-specific PTS IID | + |
| SSU05_1781 | 3.7780 | 0.0163 | ManO | + |
| SSU05_1907 | 3.9179 | 0.0175 | ABC-type sugar transport system, ATPase component | + |
| SSU05_1919 | 2.0474 | 0.0498 | hypothetical protein SSU05_1919 |  |
| SSU05_1921 | 12.3702 | 0.0062 | putative alpha-1,2-mannosidase | + |
| SSU05_1933 | 2.5113 | 0.0474 | transcriptional regulator |  |
| SSU05_2062 | 2.4824 | 0.0464 | phosphotransferase system, galactitol-specific IIB component |  |
| SSU05_2064 | 13.0276 | 0.0474 | Type II secretory pathway, pullulanase PulA and related glycosidases |  |
| SSU05_2065 | 9.8921 | 0.0378 | Type II secretory pathway, pullulanase PulA and related glycosidases |  |
| SSU05_2071 | 7.8641 | 0.0036 | phosphotransferase system cellobiose-specific component IIC | + |
| SSU05_2072 | 12.1414 | 0.0025 | hypothetical protein SSU05_2072 | + |
| SSU05_2073 | 11.8975 | 0.0004 | phosphotransferase system cellobiose-specific component IIA |  |
| SSU05_2074 | 9.5425 | 0.0054 | phosphotransferase system cellobiose-specific component IIA | + |
| SSU05_2075 | 11.7880 | 0.0017 | phosphotransferase system cellobiose-specific component IIB | + |
| SSU05_2076 | 8.4070 | 0.0084 | transcriptional antiterminator | + |
| SSU05_2077 | 6.8119 | 0.0178 | hypothetical protein SSU05_2077 |  |
| SSU05_2079 | 3.7095 | 0.0156 | alpha-galactosidase/6-phospho-beta-glucosidase |  |
| SSU05_2131 | 8.7448 | 0.0277 | 4-alpha-glucanotransferase |  |
| SSU05_2132 | 7.3067 | 0.0277 | 4-alpha-glucanotransferase |  |
| SSU05_2133 | 5.7551 | 0.0245 | ABC transporter substrate-binding protein - maltose/maltodextrin |  |
| SSU05_2134 | 5.0599 | 0.0218 | ABC-type sugar transport system, permease component | + |
| SSU05_2135 | 6.8496 | 0.0189 | ABC-type maltose transport system, permease component |  |
| SSU05_2136 | 3.8521 | 0.0156 | integral membrane protein | + |
| SSU05_2137 | 2.8833 | 0.0062 | transcriptional regulator | + |
| SSU05_2138 | 3.1889 | 0.0445 | Type II secretory pathway, pullulanase PulA and related glycosidases | + |
| SSU05_2142 | 3.2464 | 0.0212 | acyltransferase | + |
| SSU05_2143 | 3.6572 | 0.0175 | oxidoreductase | + |
| SSU05_2145 | 2.7333 | 0.0114 | glucocerebrosidase |  |
| SSU05_2146 | 2.7188 | 0.0282 | hypothetical protein SSU05_2146 |  |
| SSU05_2147 | 2.4013 | 0.0292 | beta-glucosidase |  |

* “+” indicates that the genes with increased expression levels in Δ*hp0197* were also up-regulated in Δ*ccpa.*
