# Supplementary material for: HP0197 Contributes to CPS Synthesis and the Virulence of Streptococcus suis via CcpA
Source: PLoS One. 2012 Nov 30;7(11):e50987. doi: 10.1371/journal.pone.0050987 (PMC3511442; doi:10.1371/journal.pone.0050987)
Supplement: Table S3 — Differential expression levels of proteins in Δ hp0197 compared to WT as identified by 2-DE/MS. (DOC) [file pone.0050987.s005.doc]

**Table S3. Differential expression levels of proteins in Δ*hp0197* compared to WT as** identified by 2-DE/ MS

| Spot No. | Identified protein | NCBI accession No. | Theoretical Mr/pI | MOWSE score | No. of peptides matched | No. of peptides not matched | Coverage (%) |
| --- | --- | --- | --- | --- | --- | --- | --- |
| 1 | trigger factor | 146317984 | 46.7/4.48 | 37 | 10 | 35 | 27% |
| 2 | L-lactate dehydrogenase | 146318730 | 35.4/5.05 | 107 | 14 | 25 | 43% |
| 3 | ribosome-associated protein Y (PSrp-1) | 146318094 | 22.3/5.49 | 68 | 8 | 13 | 39% |
| 4 | trigger factor | 146317984 | 46.7/4.48 | 37 | 8 | 17 | 20% |
| 5 | (acyl-carrier-protein) S-malonyltransferase | 146319458 | 33.7/4.58 | 78 | 12 | 35 | 36% |
| 6 | ADP-glucose pyrophosphorylase | 146318667 | 41.4/5.01 | 55 | 12 | 35 | 30% |
| 7 | putative PTS system,mannose-specific component IIAB | 146319432 | 35.3/4.95 | 49 | 10 | 38 | 36% |
| 8 | putative PTS system, mannose-specific component IIAB | 146319432 | 35.3/4.95 | 74 | 13 | 37 | 57% |
| 9 | putative PTS system, mannose-specific component IIAB | 146319432 | 35.3/4.95 | 72 | 14 | 35 | 49% |
| 10 | 3-oxoacyl-(acyl carrier protein) synthase II | 146319456 | 43.9/5.19 | 37 | 7 | 22 | 28% |
| 11 | 3-oxoacyl-(acyl carrier protein) synthase II | 146319456 | 43.9/5.19 | 40 | 5 | 9 | 16% |
| 12 | ornithine carbamoyltransferase | 146318280 | 37.9/5.26 | 50 | 8 | 16 | 23% |
| 13 | ribosome-associated protein Y (PSrp-1) | 146318094 | 22.3/5.49 | 121 | 15 | 35 | 61% |
| 14 | ribosome-associated protein Y (PSrp-1) | 146318094 | 22.3/5.49 | 103 | 14 | 49 | 58% |
| 15 | ABC-type sugar transport system, ATPase component | 146319561 | 42.4/5.91 | 111 | 18 | 23 | 46% |
| 16 | ABC-type sugar transport system, ATPase component | 146319561 | 42.2/5.91 | 38 | 8 | 18 | 22% |
