# Supplementary material for: HP0197 Contributes to CPS Synthesis and the Virulence of Streptococcus suis via CcpA
Source: PLoS One. 2012 Nov 30;7(11):e50987. doi: 10.1371/journal.pone.0050987 (PMC3511442; doi:10.1371/journal.pone.0050987)
Supplement: Table S4 — Primer sequences used for this study. (DOC) [file pone.0050987.s006.doc]

**Table S4. Primer sequences used for this study**

| Gene | Primer sequence |
| --- | --- |
| HP0197L-1 | Forward: 5- ATTGAATTCGGTTCAGGTGGTTTCTATGG (EcoRI) |
| HP0197L-2 | Reverse: 5- CCATGGATCCAAGTCCTAATCATATTTTA (BamHI) |
| HP0197R-1 | Forward: 5- TAAGTCGACATGAAATAATAAAGAGGCAGGGCGG (Sal I) |
| HP0197R-2 | Reverse: 5- AAGAAGCTTTCTAGACAACGGTCGTTACCATGCC (Hind III) |
| EGFP-1 | Forward: 5- AAAGGATCCATGGTGAGCAAGGGCGAGGA (BamHI) |
| EGFP-2 | Reverse: 5- AAAGTCGACTTACTTGTACAGCTCGTCCATGCCG (Sal I) |
| HP0197C-1 | Forward: 5- AAAGCATGCTGCAAGGTGTTATCATCC (SphI) |
| HP0197C-2 | Reverse: 5- CCCCGAATTCAGGTTTTGGAAACCT (EcoRI) |
| HP0197-P1 | Forward: 5- TCAACCGTGTGGTGCAAGGTGTTATCA |
| HP0197-P2 | Reverse: 5- CTAGTTTCAAAGGTTTTGGAAACCT |
| HPrF-L1 | Forward: 5- TATGAATTCGATGCTCTTGCGGCTCA (EcoRI) |
| HPrF-L2^*^ | Reverse: 5- TGAAGCCTTGTCATCGTCGTCTTTGTAGTCCATATTTGGATTCTC |
| HPrF-R1^*^ | Forward: 5- AATATGGACTACAAAGACGACGATGACAAGGCTTCAAAAGACTTCCACAT |
| HPrF-R2 | Reverse: 5- CAAGGATCCTGCGATAACGATTGACT (BamHI) |

^*^ The shaded sequence indicates the coding sequence of the Flag-tag.
