# Supplementary material for: HP0197 Contributes to CPS Synthesis and the Virulence of Streptococcus suis via CcpA
Source: PLoS One. 2012 Nov 30;7(11):e50987. doi: 10.1371/journal.pone.0050987 (PMC3511442; doi:10.1371/journal.pone.0050987)
Supplement: Table S5 — Primer sequences used for qRT-PCR. (DOC) [file pone.0050987.s007.doc]

**Table S5. Primer sequences used for qRT-PCR**

| Gene | Primer sequence used for Real-time PCR |
| --- | --- |
| transcriptional regulator（SSU05_1372） | Forward: 5’-AGTATAAATACGAGGAAGGC  Reverse: 5’-GTGGCTGATTGATAGAGG |
| Subtilisin-like serine protease（SSU05_0812） | Forward: 5’- GCACTGTAACGCCTCCAC  Reverse: 5’- AGCCCAATAATGACACCAAG |
| CPS2J ( SSU05_0573) | Forward: 5’-TTTGGAATACGCAGAGCA  Reverse: 5’- CAAGTAACCCTCCCGACA |
| Putative effector of murein hydrolase LrgA ( SSU05_0265) | Forward: 5’- CTGGCTAACATGACCATT  Reverse: 5’- TGTAGTCGCCTTCAAACT |
| transcriptional regulator （SSU05_1933） | Forward: 5’-GGAATTAGTTATAGCGGAATG  Reverse: 5’-GCAAGTCGGTTAGGAAAA |
| transcriptional regulator（SSU05_0167） | Forward: 5’-CTGCCTCATTGACTGTTC  Reverse: 5’-GATGTTCATTTGCTCCTT |
| transcriptional antiterminator（SSU05_2076） | Forward: 5’- CTACGCTTCATTCCATTT  Reverse: 5’- TTTAGCATCCCGACACTC |
| transcriptional regulator（SSU05_2137） | Forward: 5’- TGCCAGATGACTCGGATGT  Reverse: 5’-TCGTCGGGCTTGGAATAG |
| transcriptional antiterminator（SSU05_1039） | Forward: 5’-TAACGCTGCCCTGGTGAA  Reverse: 5’-CGTGTAGGTTGCCGAAAT |
| hypothetical protein SSU05_0469 | Forward: 5’-TTATTTAATCCTCGCCATCA  Reverse: 5’-TACCAAGGCAATCGCTAA |
| Galactokinase ( SSU05_0360) | Forward: 5’-CTACCTTTGGTCGTCTGG  Reverse: 5’-TGTGGACAATGGCAATAC |
| Phosphotransferase system IIC component, glucose/maltose/N-acetylglucosamine-specific ( SSU05_1401) | Forward: 5’- GCTTTGGGCAGCAGTTTA  Reverse: 5’-GAGTCGCACAGGCAGTCA |
| SSU98_0195 | Forward: 5’- AGGCTGATGTTGTGGATG  Reverse: 5’-GTTGACGGCTTGGATTGT |
| SSU98_0196 | Forward: 5’- GGCTGAAGGAGATGCTGA  Reverse: 5’-ACGAGAAGACGAGGAGGG |
| SSU98_0197 | Forward: 5’- ACTCTGCACCTAAGACAGC  Reverse: 5’-TTGCGACAACTTTACCAT |
| SSU98_0198 | Forward: 5’- CCTCCGCAATAAAGCAGC  Reverse: 5’-TGTGAGCCGTGGGTAAAG |
| SSU98_0199 | Forward: 5’- AATCCTTCCACCTGTTCC  Reverse: 5’-TCCTTACCTTCTGCTCCA |
| 16S rRNA | Forward: 5- GTTGCGAACGGGTGAGTAA  Reverse: 5- TCTCAGGTCGGCTATGTATCG |
